# Supplementary figures and images for: Ecological and life history traits are associated with Ross River virus infection among sylvatic mammals in Australia
Source: BMC Ecol. 2019 Jan 15;19:2. doi: 10.1186/s12898-019-0220-5 (PMC6334474; doi:10.1186/s12898-019-0220-5)

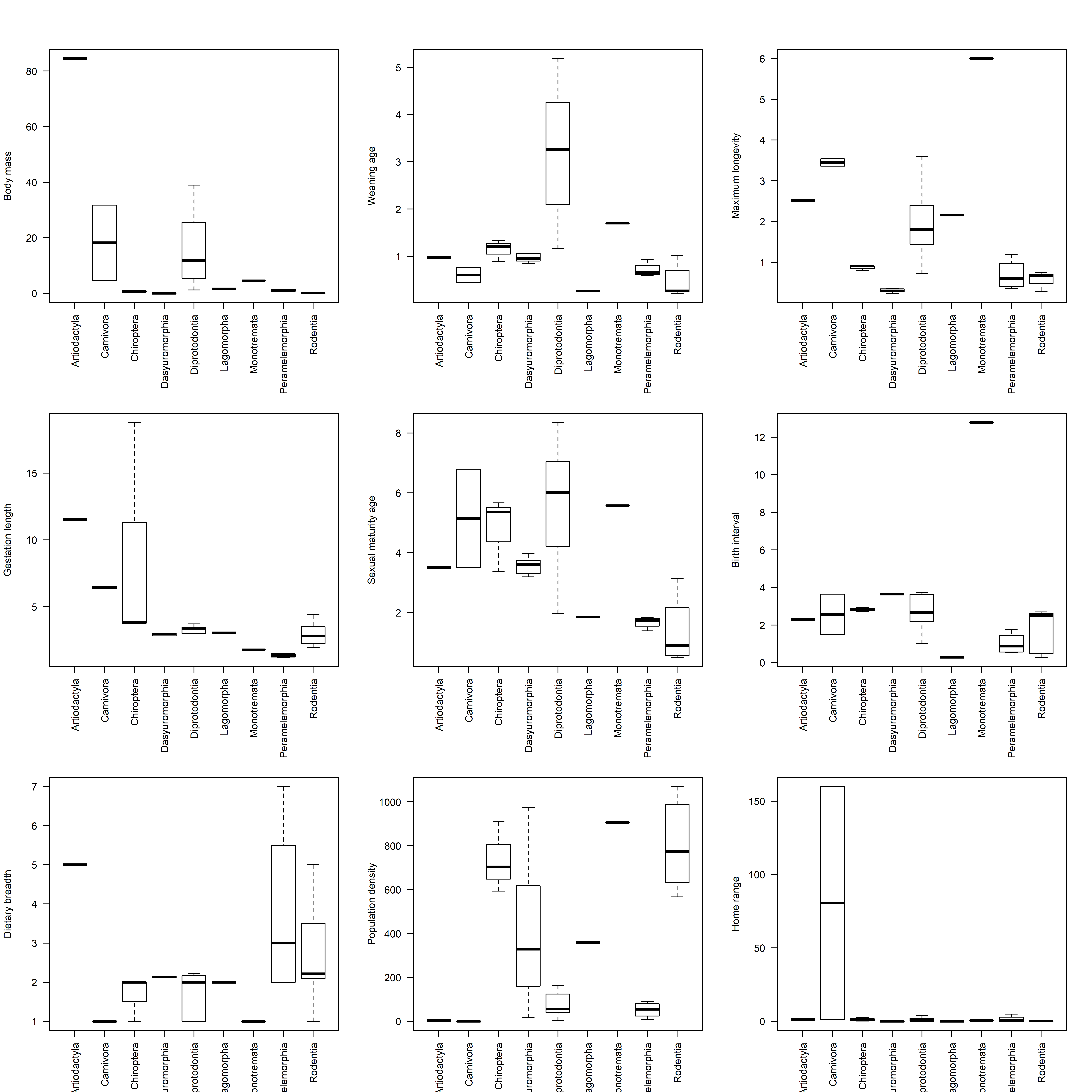

Supplement: Supplementary file 3 — Additional file 3. Boxplots of species traits by taxonomic order. [file 12898_2019_220_MOESM3_ESM.tiff]
